# Impact of Work Stress and Job Burnout on Turnover Intentions among Hotel Employees

**DOI:** 10.3390/ijerph19159724

**Published:** 2022-08-07

**Authors:** Wagih Salama, Ahmed Hassan Abdou, Shaimaa Abo Khanger Mohamed, Hossam Said Shehata

**Affiliations:** 1 Department of Social Studies, College of Arts, King Faisal University, P.O. Box 380, Al Ahsa 31982, Saudi Arabia; 2 Department of Hotel Studies, High Institute of Tourism and Hotels, Ismailia 41511, Egypt; 3 Faculty of Tourism and Hotels, Mansoura University, Mansoura 35516, Egypt; 4 Faculty of Tourism and Hotels, Alexandria University, Alexandria 21526, Egypt

**Keywords:** work stress, job burnout, turnover, intentions

## Abstract

This research aims primarily to shed light on the impact of work stress and job burnout on employees’ turnover intention in the hotel industry. Furthermore, it aims to identify the effect of work stress on job burnout besides examining the potential mediating role of job burnout in the relationship between work stress and employees’ turnover intentions in Egyptian hotels. For achieving this aim, the questionnaire was designed for Egyptian hotel employees and structured to cover four key parts: (1) demographic characteristics of employees of hotels, (2) work stress items, (3) job burnout items, and (4) turnover intentions. Structural equation modeling (SEM) results were obtained using AMOS software, IBM, version 24. The results indicate that job burnout partially mediates the relationship between work stress and turnover intentions. To be more specific, work stress has a significant positive effect on the turnover intention (β = +0.40, *p* < 0.01), and a significant positive effect on job burnout (β = +0.43 *p* < 0.01). Thus, there exists a strong positive association between work stress and turnover intentions as well as a positive association between job burnout and work stress. The findings of this study would help policymakers, hotel managers as well as practitioners to formulate policies for lessening the work stress, job burnout, and turnover intentions among hotel employees.

## 1. Introduction

Employee turnover has given rise to a challenge for the hotel organizations as they had always shown their concerns about providing the quality service to their clients [1]. The hotel industry is experiencing the labor shortages in the developed countries as well as the emerging markets [2]. In the hospitality industry, the entire turnover rate was found to be 27.6 percent in 2014 alone, in China [3], drawing the attention of the industry managers and academics [4]. Consequently, bringing down the turnover rate has become the top priority for the industry.

Hotel employees are instrumental in building positive customer experiences that are vital elements of the customer satisfaction as well as the evaluation of the service quality [5,6]. Although technological development contributes to the development of employees’ performance and capabilities, this transformation is capable of creating the nerve-wracking working conditions, where almost every employee is put under persistent observation/monitoring [7]. Duraisingam et al. [8] suggest that intense stress at the workplace adversely impacts the employees and lessens their association with the work, consequently affecting turnover intention. Hotel employees also face a lot of provocations/challenges while accomplishing their tasks/jobs [9]. Experiencing a substantial amount of work, regular variations in conditions, dearth of the feedback about performance, and truncated remunerations, they grow upset as well as exhausted very easily, which sequentially impacts their behavior adversely and might result in their resignation from the job [10].

One precarious challenge being faced by the hotel employees is work stress. Extended time of operation, diverse behaviors, a high demand/low resource job model, and miniature feedback give rise to high levels of burnout and stress [11]. With the passage of time, burnout and work stress pave the way to a low level of job performance as well as extraordinary turnover intention. Staff work pressures have captivated scholars over the previous two decades, but research studies have primarily focused on other areas, for instance accounting, sales, and nursing [12]. The hotel sector, although seen as more stressful, has attracted less attention [13].

Upon surpassing a threshold, the stress brings about the behavioral and psychological reactions for defense [14]. If hotels make available a support system that facilitates workers in relieving the stress, diminishing the deleterious emotions, and adapting to hotel establishments, the workers are expected to demonstrate higher loyalty and performance, consequently bringing down the turnover. Moreover, various studies have explored the associations among workplace characteristics, job, organizational environment, turnover intention, and self-styled support [14].

Job burnout is without a doubt a precarious issue calling for extensive attention from researchers and managers. It has been associated with a variety of adverse retorts to the profession/job, encompassing squat organizational commitment, job discontentment, and extraordinary profession/job turnover intention [15]. Several studies [16,17,18] have advocated that burnout gives rise to a considerable cost for workers as well as organizations on account of extraordinary reduced productivity, absenteeism, and job turnover. Job burnout serves as one of the most imperative predictors of turnover intention and job satisfaction.

Most studies have investigated the impact of work stress on turnover intentions. Moreover, there are studies that have investigated job burnout and its effect on turnover intentions. However, there are no prior studies that dealt with the association between work turnover and work stress and the facilitating role of job burnout. In this research study, we aim at exploring the effect of work stress and job burnout on employees’ turnover intention in the hotel industry besides identifying the adverse impact of work stress on job burnout.

### 1.1. Work Stress

Work stress is perceived to be among the phenomenal workplace health perils in the entire world for employees. The word stress refers to force or pressure on the individual caused by higher authorities [19]. Job stress has been viewed as incapability, an adverse emotional state, burden, response to job stressors, psychological state, and the trait dependence [20]. Occupational stress means the incompetence to tackle the challenge or pressure as a result of the job owing to a pitiable fit amongst the capability of the workforce and the job conditions and essentials [19].

Causes of work stress can be divided into two categories, namely internal causes and external causes. The internal causes implicate an individual’s mindset and approach, etc. They stem from within the person and bring about stress. They hinge on the perception of any individual. Even if no threat exists in the surrounding, a person might experience an intimidating situation or person and might become fatigued [21]. External causes encompass numerous external factors inside an organization that adversely impact the performance of an individual in an organization. They encompass control at work, working hours, job insecurity, managerial style, overload, and underload. Occasionally, the situations calling for the behavioral alterations might put any employee under stress [21].

The psychosocial risks caused by work stress can be classified into job insecurity, labor intensification, and imbalance in the work and personal life. The businesses are unable to continue their contracts, leading to a decline in jobs, and bringing about a sense of insecurity as well as anxiety among the employees. They must handle a higher day-to-day workload and work pressure on a daily basis. The pressure at work, greater workload, and insecure work may give rise to the problems which the workforces hold over into their private lives [22]. The work stress results in adverse consequences. Loads of behavioral glitches are the result of work-related stress, which encompasses disagreeable interactions among coworkers, increased rate of absence, and steady loss of morale [23].

Work stress is divided into two key structural dimensions, namely role ambiguity and role conflict. Role conflict spells out that when persons experience two or more expectations about their role, they are unable to fulfill both expectations simultaneously. Role ambiguity, alternatively, denotes the feelings of the employees when they are undecided or lack an appropriate mastery of their role and are unable to attain vibrant role expectations at work [24].

### 1.2. Job Burnout

Over the past few years, interest in burnout has increased as we have begun to understand the significant negative impact it has on the employees in the work environment [25]. Workplace burnout is, in fact, an ultimate calamity in the psychosomatic of the people acquaintances at work. Stress originates from environmental plus internal demands which adversely impact psychosomatic well-being. Psychosocial stressors that contribute to burnout at the workplace take account of extended working times, job uncertainty, higher workloads, poorer scenarios for salary as well as promotion, vague project roles, and time and budget pressures which contribute to errors and compromise on the standards of ethics and quality. Burnout can affect engagement at the workplace [26]. Burnout is a type of stress response that often appears in people who have direct and intense contact with others, whether they are students, clients, or guests. It arises when an individual tries to accomplish too many tasks in a short time due to unrealistic deadlines, too many projects, and meetings. While stress is not bad at times, everyone has his/her limits. Once you cross those limits, burnout is more likely to set in [27].

Workplace burnout leads to loss of productivity and employee turnover. Burnout affects the human system, thus affecting productivity and performance [28,29,30]. Burnout describes a particular type of emotional depletion with tight work, lack of commitment, and loss of motivation in young volunteers with high commitment within the work environment [31]. Maslach referred to this as the phenomenon of indifference and lack of respect for the organization’s clients [32]. 

Job burnout is, as a matter of fact, a psychosomatic syndrome associated with stress at the place of work [32]. Job burnout is, in reality, a type of mental fatigue accompanied by mental stress related to the job and work atmosphere. It is also a delayed response to factors causing chronic interpersonal stress plus mobility in the field of occupations which are likely to be seen in relieving and counseling occupations, responsibilities, and duties of these types of jobs. Job burnout is, actually, one of the foremost reverberations of work stress [33,34,35].

### 1.3. Impact of Job Burnout

Many health and psychological problems for workers result from job burnout, such as job dissatisfaction, low production quality, and work-related factors such as constantly changing expectations, work pressure without an outlet for support, and conflicting job roles that can cause job burnout syndrome [36,37]. Personality traits such as work orientation, need for assertiveness, and idealism can increase the risk of job burnout [38]. Maslach asserted that job burnout is, as a matter of fact, a rejoinder to personal and emotive stressors at the occupation/job, resulting in negative feelings such as dearth of accomplishment, low productivity at work, and inefficiency [39,40,41]. Emotional exhaustion, ranging from mild boredom to severe depression, unemotional treatment of people, and a dearth of a sense of individual completion are seen as the main causes of job burnout [42,43].

Job burnout means a condition that pops up due to the recurrent exposure to stressful situations at work that cause physical, mental, and emotional exhaustion. Job burnout continues to adversely affect workers in many organizations [44], which results in an impact on the efficacy of the institutions besides bringing about unacceptable effects on the employees. Risk factors associated with the job burnout encompass lack of support by the organization, lack of motivation, dearth of clarification, incompetence, increased responsibilities, and unrealistic expectations [45]. It also includes manifestations of controlling interpersonal relationships and withdrawal symptoms, family problems, health issues, and low performance. For this reason, all managers must implement appropriate strategies which will facilitate them in making an advantageous workstation atmosphere to protect the employees from going through the issues linked to burnout [37].

Job burnout increases alcohol addiction, which can often lead to violence and aggression within the work environment. Reasonably, once employees are content with their profession/jobs and feel extra efficient, they become able to voluntarily assist others in their related work. Similarly, when they find themselves less capable and less skillful at their work, they perceive that they are unable to control their irritability, and exhaustion causes mood swings that lead to mistreatment of others [46]. Unfortunately, there is little interest in the affiliation between the mistreatment of co-workers and job burnout, and sabotage from poor work environments [36].

Roy [41], Beheshtifar and Omidvar [47], Shah et al. [48], and Elçi1 et al. [49] referred to a model of the Maslach Burnout Inventory General Survey (MBI-GS) that was developed by Maslach and Jackson in 1996. According to this model, there exist three core facets of burnout, namely depersonalization, reduced personal accomplishments, and emotional exhaustion. The burnout occurs as workers become frustrated with their occupation/jobs and less concerned about their customers, consequently culminating in progressively adverse work-related insolence. It is associated with the forfeiture of resources and personality characteristics. It envisages numerous adverse employee-related abilities, such as absenteeism and job turnover. Furthermore, it has been associated with adverse mental health aftermaths such as depression, poor sleep, and the use of alcohol or drugs [40].

### 1.4. Turnover Intention

Turnover is viewed as the movement of employees outside the boundaries of the organization [50]. Employee turnover denotes the phenomenon of employees’ saying goodbye to an organization willingly. The decision of an employee to say goodbye to an organization is exorbitant for the organization as well as the individual [37]. Turnover intention refers to the chances that a person will leave the current job within a short period of time. In simple words, it is the employee’s intention to change the job [51]. The turnover intention may be explicated as an intention of saying goodbye to a job. High turnover is generally explicated as bad, and it is presumed expensive as it threatens quality. Another negative is the endless need for hiring and training new employees. In the healthcare sector, it is expected to have an adverse impact on fulfilling the needs of the customers plus offering a satisfactory service [52]. Three fundamental components are generally given due consideration while computing turnover costs of the employee, encompassing training costs, replacement costs, and separation costs. Labor turnover was seen as a two-dimensional concept, divided into voluntary and involuntary turnover, between an individual leaving a job and joining another workplace [53,54]. The turnover intentions were weighed with the help of an adapted three-item measure scale on the basis of Abdu [55]. These three items are as follows: (1) At present, I am surely considering resigning from my current employment in the resort; (2) Perhaps I will make an effort to find a new occupation within a year; (3) If I have a choice of choosing again, I will opt for working in another profession [40,56].

### 1.5. The Association between Work Stress and Turnover Intentions in Hotels

Liu et al. [57] mentioned that work stress had a positive and ancillary impact on the turnover intentions of the employees. Job satisfaction weakens the impact of work stress on turnover and indicated only a univariate linkage between work stress and turnover intentions. The rate of work turnover intentions rises because of the high work stress that results from the workload and negative emotions at work.

Ahn and Chaoyu [58] and Zahra et al. [59] laid emphasis on the existence of a relationship between work stress and the rate of work turnover from the perspective of organizational justice. They explained the effect of organizational justice on the unsteadiness of the negative effects that increase the rate of work turnover, and they revealed a direct connection between the intention of turnover and work pressure, with the presence of satisfaction as a mediating variable between the three variables. Prasetio et al. [60] supported a significant positive connection between turnover intentions and work stress in hotels. Hotel employees who have lower stress levels will exhibit a lower level of intention to quit. Moreover, Omar [24] revealed a positive association between intention to leave and job stress. The extraordinary stress of the employees increases their aspiration to leave the workstation [61]. Though every employee aspires to leave the work at diverse levels, work stress is an imperative factor behind the intention to leave, and it increases the probability of resigning from the work. Huang et al. [62] referred to the effect of avoidance, social support, and problem-solving as the managing strategies for turnover intentions and occupational stress among the employees of a hotel. They found that occupational stress was positively associated with the intentions of the hotel employees regarding leaving the job. The study also elucidated occupational stress’s role as an imperative facilitator in the association between the managing strategies.

### 1.6. The Relationship between Work Stress and Job Burnout

Li [63] and Firouzbakhsh [64] indicated a correlation between job burnout and work stress, showing that jobs in the healthcare sector are exceedingly stressful, the work atmosphere is pathetic, and occupational experience, work relationships, work responsibilities, and work overload add to the occupational tension [65,66]. The psychological aspect has turned out to be a more and more obvious social issue among medical workers. Thus, it is understandable that extended working hours and performing frequent shift work result in anxiety, burnout, tension, negative emotions, and depression along with physical tiredness, which further emanant a decline in quality of life and working ability [67,68].

Other researchers persuaded burnout amongst correctional officers and prison caseworkers [69]. They focused on the effect of correctional officers’ burnout and job stress, the effect of organizational commitment, job satisfaction, and job stress on burnout [70], and the effect of cynicism and depersonalization on burnout [71].

Concerning job burnout, the employees in five-star hotels informed that they occasionally feel burned out with their work just due to the work stress. They elucidated that they become fatigued as a consequence of excessive work demands. Various employees remarked that work stress has resulted in burnout of the employees. Job burnout and work stress are positively correlated. It is hypothesized that burnout is positively correlated with JS amongst hospitality employees [72]. Hu et al. [73] indicated that applying direct work strategies in dealing with work stress for some hospitality sector supervisors reduces the possibility of job burnout. Wen et al. [74] stated that there are four facets of role stress: qualitative overload, quantitative overload, ambiguity, and conflict. Role ambiguity and role conflict are found to be expressively associated with burnout. Ambiguity has an adverse impact on the employees. The study also revealed that role conflict is a vital conjecturer of emotional exhaustion. Moreover, role ambiguity is positively associated with the dearth of self-perception and turnover intention of front-line hotel employees. Furthermore, job stressors have been found to be positively associated with emotional overtiredness amongst hotel employees.

### 1.7. The Relationship between Job Burnout and Turnover Intention in Hotels

Job burnout causes negative effects on employees—i.e., negative attitudes toward the job and indifference to customer service [75]. Job burnout is a syndrome that occurs through interaction with various personal, environmental, and professional factors [76]. The increased competition and the development of human resource techniques have facilitated the organizations in sustaining capable/competent employees besides enabling them to keep their performance high. Nonetheless, the organizations are at all times scared of mislaying their human capital as each organization bears a lot of expenses for educating, culturing, and preparing its staff in attaining prime efficiency and productivity; the organization experiences mislaying experiences and skills by losing its critical and valuable workforce, for which the organization has put in years of effort [34].

As organizations are well aware of the efficacious factors as well as the causes behind employees’ leaving the organization, they employ various effective policies and techniques for sustaining the efficacious human resources beforehand. Hence, studies are needed that explore the effect of conflict in life and work and job burnout [77]. Imperative research studies were conducted on the existence of burnout to increase the absenteeism rates of the employee besides exploring the statistically significant correlations between employee absenteeism and the dimensions of job burnout. These studies revealed that the dimensions of job burnout were found to be associated with absenteeism attitudes and behaviors [45]. Han [78] indicated that the customer rudeness and its relationships explicit to the workplace environment of the restaurant that give rise to job burnout for the workforces in addition to its impact on the intent of work turnover among frontline employees in the restaurants. These studies also revealed that organizational as well as supervisory support has noteworthy cross-level interaction impacts on the association between burnout and customer rudeness besides a substantial effect on the turnover intentions of the employee.

Rahim and Cosby [79] explored the presence of a positive association between job burnout and the rate of work turnover. They found out that job burnout arbitrates the association between turnover intention and workplace rudeness. Moreover, workplace rudeness was found to be adversely associated with job performance. To put it another way, participants undergoing advanced levels of rudeness stated superior levels of the job burnout and consequently augmented turnover intention and lesser levels of job performance. Rahim and Cosby [79] showed that there exists a direct relationship, linking burnout and turnover intention about the ancillary effect of burnout on the turnover intention with work engagement acting as the partial mediator. Kartono and Hilmiana [80] showed that job burnout positively impacts the turnover intention. This is contemplated in the job burnout indicators, namely declining work performance, depersonalization, and exhaustion [75]. Exhaustion is permeated by boredom and the sense of too much workload, whereas depersonalization is permeated by selfishness (always believing that he or she is far better than others). In addition, declining work performance is permeated by low productivity and self-efficacy, which are the factors responsible for the turnover intention in any company.

Bayer [81] explained that the employee should invest more in physical and mental standards besides making more effort in case of difficulties in achieving them. For employees, workload might be a sweet burden plus a disturbing nightmare; an appropriate workload could inspire employees continuously for mastering their skills, enhancing their confidence, and promoting their work performance [82]. Kim and Stoner [83] suggest that the emotional exhaustion subscale poses the utmost effect on the turnover intention. An average decrease in employee turnover results from an increase in employee job satisfaction. The lower level of job satisfaction of the nurses taking care of the patients diagnosed with COVID-19 has been found to be hurting their burnout and turnover intention [84].

### 1.8. The Mediating Role of Job Burnout in the Relationship between Work Stress and Turnover Intentions

Thomas [53] emphasized that the employee will show different reactions when workloads are excessive. He stressed that job burnout is amongst the causes of increased work turnover intentions. The employee turnover intention arises when the individual feels a lot of work pressure and job dissatisfaction. There exist parallels between turnover intention and burnout besides remarkable impacts of burnout on turnover intention [18] because an employee experiencing higher burnout would display greater turnover intention [54]. Shanafelt et al. [85] determined the notably positive parallels between turnover intention and burnout. Shreffler et al. [86] indicated the considerably positive impacts of burnout on turnover intention along with the facilitating effects of emotional exhaustion, dehumanization, and diminished personal accomplishment.

**Figure 1:**
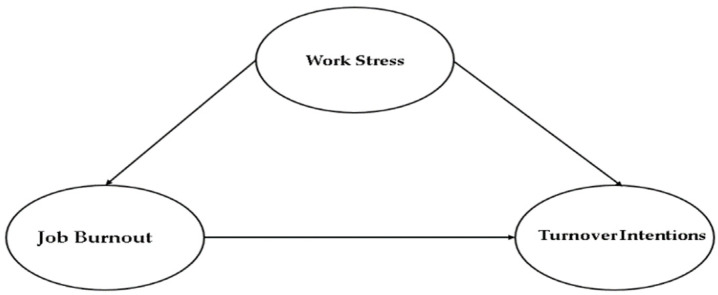
The research conceptual model.

Qattan [87] talked about a positive association between burnout and levels of work-related stress among hospital nurses serving in all three kinds of hospitals in Saudi Arabia. On the other hand, there exists a very feeble association between job performance and work-related stress amongst private hospital (International Medical Centre) nurses as compared to the métier of this association witnessed in public hospitals. Chiang et al. [88] referred to a positive association among work stress, job burnout, and employee turnover intentions of attendants of the hotel rooms. It revealed that job stress results in greater job burnout, which sequentially impacts their turnover intentions. Furthermore, organizational commitment and internal marketing considerably arbitrate job burnout and job stress. Jung et al. [89] also confirmed this positive relationship through the study which refers to understanding interconnections among turnover intention in a deluxe hotel, burnout, and perception of role stress of the culinary employees. It presented a positive association between burnout and perceptions of role stress of the employees. Participants with a greater level of burnout were more likely to leave their work/position. Moreover, moderating effects associated with the tenure in the causal relationships were found between turnover intent and burnout of the employees (Figure 1).

Based on the preceding literature review, we assumed the following:

**H1.**  *Work stress significantly affects employees’ turnover intentions in the hotels.*

**H2.**  *Work stress significantly affects job burnout in the hotels.*

**H3.**  *Job burnout significantly affects employees’ turnover intention in the hotels.*

**H4.**  *Job burnout significantly and positively mediates the relationship between work stress and employees’ turnover intentions.*

## 2. Materials and Methods

### 2.1. Measures and Instrument Development

In this research study, data was collected mainly via the self-administrated questionnaire. As a result of a wide-ranging analysis of the literature, a standardized questionnaire was developed by pinpointing valid as well as recurrently used measures. The questionnaire entails four sections. The first section handled demographic data of the participants, encompassing age, education level, gender, and marital status. The second section took account of the perceptions of the participants concerning work stress. The work stress scale developed by Rizzo et al. [90] was improved and employed for identifying the perceptions of the participants concerning work stress via role ambiguity items and role conflict with the help of a five-point Likert scale ranging from 1 = strongly disagree to 5 = strongly agree. A unidimensional scale was used. The scale entails fourteen items (i.e., “I receive an assignment without adequate resources and materials to execute it” and “The explanation is unclear of what has to be done”). A greater value of the average score replicates higher work stress declared by the participants. The internal consistency reliability (Cronbach’s alpha) for the work stress scale was found to be 0.951. The third as well as fourth sections envisioned to divulge the turnover intentions and perceptions of job burnout of the participants.

With regard to JBO, the emotional exhaustion burnout scale, as a subscale of the Maslach Burnout Inventory–General Survey (MBI-GS), suggested by Maslach and Jackson [18], validated and utilized in numerous studies, was modified and utilized to investigate JBO among the investigated hotel employees. The scale included nine items which were calculated by using a five-point Likert scale ranging from 1 = never to 5 = always. A sample item is “You feel emotionally drained from your work”. A greater value of the average score replicates greater job burnout perceived by the participants. The scale had good internal consistency (α = 0.910).

The turnover intentions were appraised by means of an improved three-item measure scale in line with Abdou et al. [55]. These items are as follows: (1) If you are given a choice to choose once again, you will decide to work in another occupation/job; (2) At present, you are surely considering saying goodbye to your current job in the hotel; (3) Perhaps I will make an effort to find a new occupation/job within next year or less. The response rate was quantified with the help of a five-point Likert scale ranging from 1 = strongly disagree to 5 = strongly agree. A greater value of the average score replicates a greater intention to leave. The internal consistency reliability (Cronbach’s alpha) about the turnover intention scale was 0.906.

The survey was originally prepared in the English language and was then translated into the native Arabic language of the participants. It was then reverse translated from Arabic to English for confirming that there existed no changes in meaning. Further, to guarantee that the study instrument quantifies the constructs set out for measuring the variables of the study, face validity of the questionnaire was confirmed by four hospitality academics who were requested to evaluate the content of the questionnaire as well as to offer any feedback. Additionally, a pilot study was carried out on a sample of 25 hotel employees, who have not been incorporated in the main sample of the study with the intent of exploring the viability of the questionnaire by testing if the questionnaire was appropriate and coherent plus if the questions were clearly understood, well-defined, and presented consistently. In line with the comments of the participants, a modification was made to the language and wordings of some statements. Even some statements were also reorganized and re-ordered.

### 2.2. Sampling and Data Collecting

As stated earlier, the foremost aim of this study is to ascertain the effect of the work stress on JBO plus turnover intentions besides exploring the probable facilitating role of JBO in the association between turnover intentions and work stress in a sample of three- and four-star hotels in Egypt. For attaining this aim, a self-administered questionnaire was developed and sent to the selected employees to ascertain their perceptions of the study constructs (work stress, JBO, and turnover intentions). The research team, using their associations with human resources managers and employees of the hotels in Egyptian destinations, requested them to participate in the field study. The convenience sampling technique is “a type of nonprobability sampling, in which people are sampled simply because they are convenient sources of data for researchers” Lavrakas [91].

Consequent to the recommendation of Hair et al. [92], a decision was made on the suitable sample size. They recommended computing the suitable sample size on the basis of the number of the explored variables. The minimum ratio (variable:sample = 1:10) is adequate. As a result, the minimum sample size essential for this study was 260 participants, wherein the total variables under exploration were 26. The sample size of 279 participants in our study was adequate. Based on the valid responses obtained from the investigated participants (279), more than three-quarters of the considered participants (77.4%, N = 216) were males and 22.6% were females. Regarding age, participants with an average age ranging from 30 to 45 years represented the greater category (52.7%, N = 147). In terms of the educational level of the participants, those who had a university degree represented 41.9% (N = 117). About their marital status, married participants were 72.8% (N = 203).

Participants were informed that taking part in the study is completely voluntary. Before taking part in this study, they were requested to sign a consent form. Because the study was using a self-administrated questionnaire, common method variances (CMV) could pose an issue. For reducing the likelihood of common method variance (CMV), the participants were guaranteed that the collected data would be kept confidential and anonymous and would be used only for the research purposes. Participants were requested to provide answers to all questions honestly, as there were no incorrect or correct responses. Further, a simple and common statistical tool (Harman’s single-factor test) was used for discovering CMV [93]. The data collection period lasted nearly two months, from January 2022 to March 2022.

### 2.3. Data Analysis

The analysis of the collected data was done with the help of SPSS v. 22 and Amos v. 24. For reflecting the respondents’ demographic data besides ascertaining their perceptions towards study constructs, descriptive statistics incorporating percentage, standard deviation, mean, and frequencies were used. Common method variance (CMV) was observed via Harman’s single factor test. The reliability and validity of the measurement items were validated by confirmatory factor analysis (CFA) and reliability analysis (Cronbach’s alpha). The average variance extracted (AVE) plus composite reliability (CR) were computed for the validation of the convergent validity. Discriminant validity on the basis of Fornell–Larcker criterion and Heterotrait–Monotrait ratio (HTMT) was also studied. Finally, structural equation modeling (SEM) with bootstrapping approach was implemented to ascertain the direction plus inter-relationships between the study hypotheses.

## 3. Results

### 3.1. Descriptive Statistics

Table 1 presents the standard deviation and mean of every construct of the research study. With regard to the work stress, the investigated participants showed their consent on the majority of the investigated items, wherein the average mean accounted for 4.02. Regarding the other constructs, they highly perceived job burnout and turnover intention. The average mean constituted 4.37 and 4.23, respectively.

### 3.2. Measurement Model

As stated earlier, the data were gathered with the help of a self-administrated questionnaire. Hence, a common method of variance/bias (CMV) was observed using Harman’s single-factor test [94]. Consequently, one component was observed to constitute only 41.01% (smaller than 50%) of the variance that divulges that CMV does not characterize a concern.

**Figure 2:**
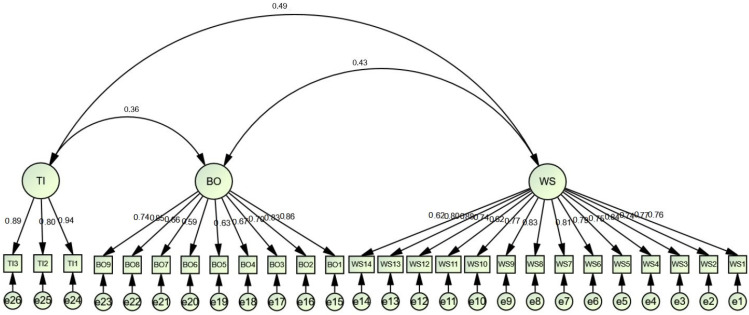
Confirmatory Factor Analysis.

For discovering the reliability and validity of the study constructs, CFA using maximum probability was administered (see Figure 2). As shown in Table 1, the values of composite reliability (CR) and Cronbach’s alpha of all latent variables surpass the recommended threshold of 0.80 [92], specifying acceptable internal reliability.

Construct validity was also studied with the help of discriminant and convergent validities [95]. Convergent validity demands a factor loading of not less than 0.50 as well as an average variance extracted (AVE) above 0.50 [96]. The factor loading of all the study objects is greater than 0.50, and the AVE of each construct was found to be above 0.50, ranging from 0.537 to 0.769, which indicates that convergent validity has been attained. In line with the criterion of Fornell–Larcker, the discriminant validity of the constructs necessitates the square root of AVE of each construct to be higher than its correlation with another construct. Data in Table 2 exemplify that the AVE’s square root of all the constructs is greater than their correlations with other ones. Furthermore, the Heterotrait–Monotrait Ratio of correlations (HTMT) proposed by Henseler et al. [66] to test discriminant validity was also utilized. In their view, discriminant validity is compromised when the HTMT value exceeds 0.85. In line with the results in Table 3, all HTMT values were less than 0.85, indicating that discriminant validity was present in all pairs of latent constructs.

Concerning the fit of the study’s model, the data presented in Table 1 indicate that it was good; x^2^/df = 3.396, *p* < 0.001, Comparative Fit Index (CFI) = 0.917, Normed Fit Index (NFI) = 0.901, Incremental Fit Index (IFI) = 0.917, Tucker–Lewis coefficient (TLI) = 0.907, Root Mean Square Residual (RMR) = 0.054, Root-Mean Square Error of Approximation (RMSEA) = 0.076.

### 3.3. Structural Equation Modeling (SEM)

**Figure 3:**
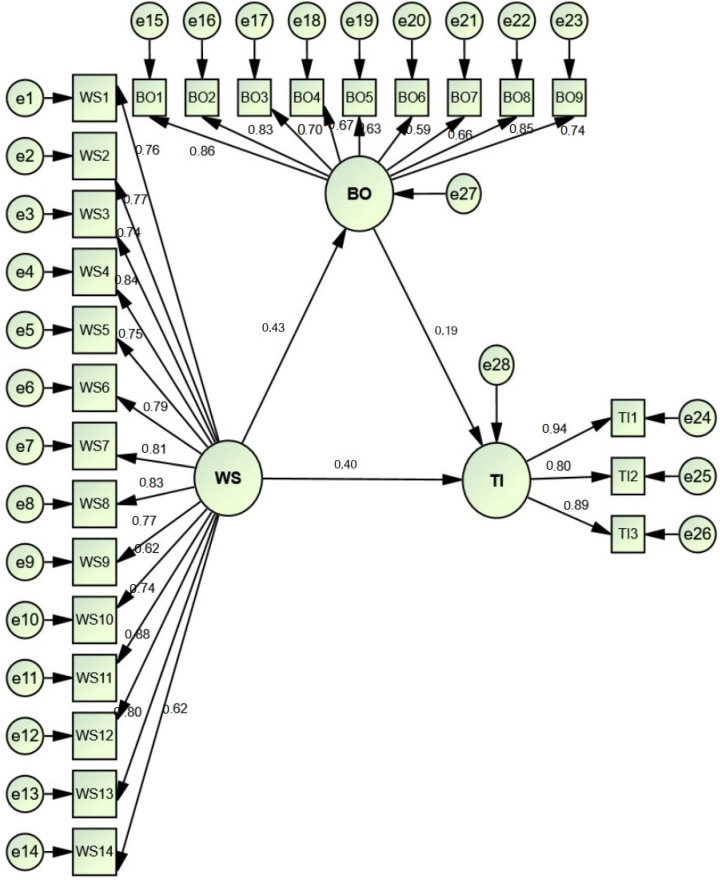
The structural model.

To determine the direction and interconnections among study hypotheses, SEM was brought into play. Data in Table 4 divulged that the fit of the research model was good as acclaimed by Hair et al. [92]. The goodness of fit indices was as follows; x^2^/df = 3.396 *p* < 0.001, CFI = 0.917, NFI = 0.901, IFI = 0.917, TLI = 0.907, RMR = 0.054, RMSEA = 0.076. The results in Table 4 and Figure 3 elucidate the direct effect of work stress on job burnout and turnover intention, and the indirect effect of work stress on turnover intention via the facilitating role of job burnout. Hypothesis 1 (Work stress significantly affects employees’ turnover intentions) is supported (β = 0.403, t-value = 7.376, *p* < 0.001). Likewise, work stress significantly and positively affects job burnout (β = 0.432, t-value = 8.223, *p* < 0.001). Hence, H2 is supported. Hypothesis 3 that anticipated that JBO significantly affects TI (β = 0.193, t-value = 3.654, *p* < 0.001) is also supported. A bootstrapping approach was utilized to validate the indirect association between turnover intention and work stress besides exploring the role that job burnout might play in it. The results in Table 4 emphasized that WS had indirect, positive, and significant effects on TI via JBO (β = 0.083, t-value = 2.452, *p* < 0.01). Henceforth, H_4_ is accepted. To analyze the arbitration effect of JBO on the association between professed WS and TI, the path was appraised using both partial and full mediation suggestions from Kelloway [98] and Zhao et al. [99]. They showed that full mediation can only be determined if the indirect impacts are significant, whereas the direct impacts are not significant; partial mediation can only be established if both paths are significant. According to the outcomes of the SEM, we can conclude that JBO partially mediates the relationship between WS and TI.

## 4. Discussion and Implications

The main objective of this research was to identify the impact of work stress on employees’ turnover intentions as well as to explore the potential mediating role of JBO in the relationship between WS and TI in a sample of three- and four-star hotels in Egyptian destinations. In line with the earlier literature, the intangible model suggested in this study hypothesized that work stress meaningfully impacts the turnover intention of the employees directly and indirectly via job burnout. Likewise, the model also assumed that work stress has a noteworthy impact on job burnout, and JBO too has a substantial impact on the turnover intention of the employees.

With regard to the association between WS and TI, the outcomes of the study revealed that WS has a significant positive effect on TI among the surveyed hotels’ employees. This outcome is consistent with the findings of most prior studies that examined the association between two constructs. Prasetio et al. [60] concluded that WS positively and significantly affects employees’ turnover intention in a privately owned hotel in Karawang, West Java. Further, this finding supports the results of the empirical study conducted by Rehman and Mubashar [100] on a sample involving 200 employees from dissimilar hotels in Lahore, Pakistan, who concluded that work stress is positively correlated with turnover intentions. This finding reinforces the empirical study outcomes adopted by Babakus et al. [101] on Turkish hotel employees suggesting that role stressors (role ambiguity and role conflict) provoke emotional exhaustion of the frontline employees. Moreover, a study conducted in seven cities in South China by Wen et al. [74] confirmed that role stress as a four-dimensional construct (i.e., quantitative overload, qualitative overload, ambiguity, and conflict) has a statistically significant effect on hotel front-line employees’ turnover intentions. From this finding, one can conclude that the greater the professed work stress, the greater the professed turnover intentions among hotel employees. Hotel employees who have lesser stress levels will exhibit a lower level of intention to quit.

Additionally, the findings of the study confirmed the causal relationship between WS and JBO. The study findings indicated that employees’ job burnout is significantly and positively affected by work stress. This finding fosters the results of previous research that argued that WS positively and significantly affects JBO in the hotel industry (i.e., Wan et al. [74]; ‘Sunny’ Hu and Cheng [73]; Chiang and Liu [88]. Among hotel supervisors in Taiwan hotels, ‘Sunny’ Hu and Cheng [73] suggested that the foremost job stress of the hotel administrators emanates from the characteristics of the assigned task and the amount of work which significantly affects employees’ job burnout. Chiang and Liu [88] examined the association between burnout and job stress among room attendants. They indicated a positive association between job stress and job burnout. The room attendants experiencing job stress do have greater job burnout. Furthermore, the outcome of this research study is in accordance with that revealed by Jung et al. [91] who investigated the relationship between WS and JBO among the Korean hotels’ culinary employees and exhibited a positive connection between burnout and the perceptions of role stress of the employees. Upon the previous finding, we confirm that WS is the main predictor of JBO among Egyptian hotel employees. The higher the perceived job stress, the greater the job burnout.

The finding of the study also indicated that JBO has a positive significant impact on TI. From this finding, it could be suggested that the greater the perceived JBO, the higher the perceived TI. This outcome is in line with the pragmatic study findings conducted by Babakus et al. [101] who confirmed the positive significant relationship between hotel frontline employees’ turnover intentions and emotional exhaustion in Turkish hotels. Among restaurants’ service frontline employees, Han et al. [78] concluded that job burnout is a key forecaster of employees’ turnover intentions. This outcome is also in agreement with Wen et al. [74] who found that burnout has a significant positive effect on employees’ turnover intention. Hence, one can conclude that the greater the experienced JBO, the greater the professed TI.

Regarding the facilitating role of job burnout in the work stress—turnover intention relationship—the finding of the study suggested that job burnout to some extent arbitrates the relationship between WS and TI. This outcome is in agreement with the outcomes of earlier studies in different contexts. Cho et al. [102] showed that emotional exhaustion to some extent arbitrates the association between job stress factors (conflict, ambiguity, and overload) and turnover intention of the employees in the airline industry. Further, in the hospital context, Tziner et al. [97] concluded that job burnout partially mediates the association between turnover intentions and work stress among hospital physicians. This outcome is at odds with Wen et al. [74] who revealed that job burnout fully arbitrates the association between turnover intention and role stress; hotel employees with the role stress do not put up resignation immediately unless they encounter higher levels of burnout. In commercial banks of Pakistan, Aqeel and Sher [103] revealed that the association between job stress and turnover intention is partially arbitrated by job burnout. As a result, it could be concluded that hotel employees experiencing prolonged or excessive job stress can eventually become emotionally exhausted, which can ultimately lead to voluntary turnover.

In essence, the findings of this research study confirm that the work stress and job burnout are the key predictors of turnover intentions of the employees. Furthermore, the blatant work stress positively and significantly impacts JBO. Additionally, the outcomes demonstrate that JBO significantly arbitrates the association between work stress and turnover intentions.

Upon the findings of the study, some pragmatic implications for practitioners of the hotel industry could be interpreted as follows. The findings of this study would help policymakers, hotel managers, and practitioners in formulating policies for diminishing work stress, job burnout, and turnover intentions among hotel employees. Hotel managers need to understand that creating a flexible and supportive work environment among hotel employees is vital. Employee retention can be enhanced by reducing work stress and providing a more enjoyable working environment. Receiving assignment work without the adequate resources and workers to execute it, receiving conflicting requirements from two or more people, having unclear or unplanned objectives, unclear explanations of what has to be done, and dividing work time improperly should be discouraged. Having adequate social and organizational support from hotel supervisors and managers is crucial. Social and organizational support from hotel executives significantly negatively affects JBO and TI and positively affects employees’ emotional engagement. Providing a supportive work environment that encourages mindfulness and resilience among hotel employees is extremely important. Practicing mindfulness aids in the self-control of thoughts, feelings, and behaviors, thereby minimizing burnout-causing emotional stress. Further, under stressful situations, resilient employees engage in active coping strategies that allow them to overcome adverse circumstances more effectively. Factors affecting the level of stress, job burnout, and turnover intentions among hotel employees should be identified.

## 5. Limitations and Further Research

In the current study, we have encountered some limitations. Firstly, this study was conducted on hotel employees serving in three- and four-star hotels in Egypt. Therefore, it would be difficult to generalize these results. These findings should apply specifically to this category of the hospitality industry. Possibly, the upcoming research may examine how employees perceive WS, JBO, and TI in other hotel types (such as luxury hotels). Secondly, as a mediator in the relation between WS and TI, the study only examined one dimension of job burnout (emotional exhaustion). In future research, the other dimensions of JBO (depersonalization and reduced personal accomplishment) may also be explored. Thirdly, the data was gathered with the help of a self-administered questionnaire, allowing the participants to provide answers to the questions consistent with their personal perceptions. Future research may provide a better understanding if it is conducted using both quantitative and qualitative methods. Fourthly, future studies may examine how JBO affects the other variables associated with behavior and attitude of the employees instead of their turnover intentions (i.e., performance, satisfaction, commitment, organizational citizenship behavior … etc.). Finally, backgrounds and consequences of work stress in other hospitality sectors (i.e., restaurants, resorts, cruises … etc.) may be examined.

## 6. Conclusions

Work stress represents the employee’s feeling in a negative emotional state towards the work environment and his inability to deal with work pressure as a result of its accumulation, resulting in a state of job burnout, which is considered one of the occupational health risks, affecting the job satisfaction rate and impacting the rate of achievement and the employee’s lack of productivity and status from imbalance, ultimately resulting in a state of boredom that reaches the severity of depression for employees in the work environment. All of these reasons lead to employees’ desire to leave work and search for other jobs in other hotels, where the intensity of the pressure is less than in their current job. The study recommends the necessity of identifying the causes of work pressures, relieving them, distributing tasks to employees equally, and observing organizational justice in hotel work, as an attempt to diminish the severity of job burnout amongst employees as well as to convince them to fit the current work environment with their living conditions so that they do not have the intentions of work turnover and the search for another job. For sustaining the competent/qualified employees at work, it will be beneficial to certify the insights that the valuable contributions of the employees are imperative for the hotel. Furthermore, the management of the hotel must take care of the employees. Even if the performance of the employees in the organization declines due to any reason, the executives must support the employees for their welfare and happiness and should permit them to pursue their work.

## Figures and Tables

**Table 1:** Reliability and Confirmatory Factor Analysis Properties.

Construct/Items	Std. Loading (CFA) ^1^	Mean (Standard Deviation)	Cronbach’s Alpha	CR ^2^	AVE ^3^
***1.*** ***Work Stress (WS)***					
WS1: You must do things that should be done in a different manner.	0.755	4.02 (0.878)	0.951	0.953	0.591
WS2: You receive an assignment devoid of the required manpower to accomplish it.	0.768				
WS3: You must defy a policy or rule in order to complete an assignment.	0.743				
WS4: You work with two or more groups that operate quite differently.	0.842				
WS5: You receive conflicting tasks from two or more people.	0.748				
WS6: You receive an assignment devoid of suitable materials and resources to accomplish it.	0.789				
WS7: You do things which are appropriate to be taken on by one person and not taken on by others.	0.813				
WS8: You work on unnecessary things.	0.828				
WS9: You have not planned clear objectives and goals for your job.	0.772				
WS10: You are aware that you have not divided your time appropriately.	0.621				
WS11: You are not aware of your responsibilities.	0.743				
WS12: You do not exactly know what is expected of you.	0.881				
WS13: It is not clear what must be done.	0.800				
WS14: You are uncertain about your authority.	0.618				
***2.*** ***Burnout (BO)***					
BO1: You feel emotionally drained from your work.	0.860	4.37 (0.620)	0.910	0.911	0.537
BO2: You feel exhausted by the end of the workday.	0.830				
BO3: You feel overtired when you wake up in the morning and have to experience another day on the job.	0.699				
BO4: Working with people of diverse nature all day is actually a tension for you.	0.674				
BO5: You feel burned out due to your work.	0.634				
BO6: You feel frustrated by your job.	0.585				
BO7: You feel you are working too hard on your job.	0.664				
BO8: Working directly with people puts too much tension on you.	0.849				
BO9: You feel like you are simply finished.	0.742				
***3.*** ***Turnover Intention (TI)***					
TI1: If you are given a choice to choose again, you will choose to work in another occupation/job.	0.939	4.23 (0.840)	0.906	0.909	0.769
TI2: At present, you are really considering giving resignation from your current job in the hotel.	0.800				
TI3: Perhaps I will make an effort to find a new job within next year or less.	0.886				

Std. Loading, (CFA) ^1^ = Standardized Factor Loading, CR ^2^ = Composite Reliability, AVE ^3^ = Average Variance Extracted. Model fit; x^2^/df = 3.396, *p* < 0.001, Comparative Fit Index (CFI) = 0.917, Normed Fit Index (NFI) = 0.901, Incremental Fit Index (IFI) = 0.917, the Tucker–Lewis coefficient (TLI) = 0.907, Root Mean Square Residual (RMR) = 0.054, Root Mean Square Error of Approximation (RMSEA) = 0.076.

**Table 2:** Constructs’ Correlation and Discriminant Validity Based on Fornell–Larcker Criterion.

Construct	1	2	3
1-Work Stress	**0.769**		
2-Job Burnout	0.432 ***	**0.733**	
3-Turnover Intention	0.487 ***	0.364 ***	**0.877**

*** *p* < 0.001 The bold diagonal values represent the square root of AVE.

**Table 3:** Discriminant Validity via HTMT.

Construct	1	2	3
1-Work Stress			
2-Job Burnout	0.480		
3-Turnover Intention	0.498	0.388	

Note: HTMT should be less than 0.85 Henseler et al. [97].

**Table 4:** Structural Parameter Estimates.

Hypothesized Path				Standardized Path Coefficients	*t*-Value	Results
H1: Work stress	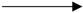	Turnover Intention		0.403	7.376 ***	Supported
H2: Work Stress	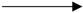	Job Burnout		0.432	8.223 ***	Supported
H3: Job Burnout	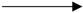	Turnover Intention		0.193	3.654 ***	Supported
H4: Work Stress	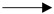 Job Burnout	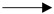	Turnover Intention	0.083	2.452 **	Supported

Note: Model fit; x^2^/df = 3.396 *p* < 0.001, CFI = 0.917, NFI = 0.901, IFI = 0.917, TLI = 0.907, RMR = 0.054, RMSEA = 0.076, *** *p* < 0.001, ** *p* < 0.01.

## Data Availability

Data are available upon request from the researchers who meet the eligibility criteria. Kindly contact the first author privately through e-mail.

## References

[1] Ahmad A., Barakbah S.M., Majdi A.K. (2021). Hotel Employees’ Burnout and Turnover Intentions. Webology.

[2] Kim S.-M., Um K.-H., Kim H.Y., Kim Y.-H. (2014). Hospital career management systems and their effects on the psychological state and career attitudes of nurses. Serv. Bus..

[3] Chen J., Wang L., Tang N. (2014). HALF THE SKY: The Moderating Role of Cultural Collectivism in Job Turnover Among Chinese Female Workers. J. Bus. Ethic.

[4] Mohsin A., Lengler J., Kumar B. (2013). Exploring the antecedents of intentions to leave the job: The case of luxury hotel staff. Int. J. Hosp. Manag..

[5] Wirtz J., Jerger C. (2016). Managing service employees: Literature review, expert opinions, and research directions. Serv. Ind. J..

[6] Elmadağ A.B., Ellinger A.E. (2017). Alleviating job stress to improve service employee work affect: The influence of rewarding. Serv. Bus..

[7] Serinikli N. (2019). İş stresinin işten ayrılma niyetine etkisinde örgütsel bağlılığın aracılık rolü. Bus. Econ. Res. J..

[8] Duraisingam V., Pidd K.J., Roche A. (2009). The impact of work stress and job satisfaction on turnover intentions: A study of Australian specialist alcohol and other drug workers. Drugs Educ. Prev. Policy.

[9] Kim S.S., Im J., Hwang J. (2015). The effects of mentoring on role stress, job attitude, and turnover intention in the hotel industry. Int. J. Hosp. Manag..

[10] Kim T.T., Paek S., Choi C.H., Lee G. (2012). Frontline service employees’ customer-related social stressors, emotional exhaustion, and service recovery performance: Customer orientation as a moderator. Serv. Bus..

[11] O’Neill J.W., Davis K. (2011). Work stress and well-being in the hotel industry. Int. J. Hosp. Manag..

[12] Kuo H.-T., Lin K.-C., Li I.-C. (2013). The mediating effects of job satisfaction on turnover intention for long-term care nurses in Taiwan. J. Nurs. Manag..

[13] Cho S., Johanson M.M., Guchait P. (2009). Employees intent to leave: A comparison of determinants of intent to leave versus intent to stay. Int. J. Hosp. Manag..

[14] Jaramillo F., Mulki J.P., Boles J.S. (2011). Workplace Stressors, Job Attitude, and Job Behaviors: Is Interpersonal Conflict the Missing Link?. J. Pers. Sell. Sales Manag..

[15] Lu A.C.C., Gursoy D. (2016). Impact of Job Burnout on Satisfaction and Turnover Intention: Do Generational Differences Matter?. J. Hosp. Tour. Res..

[16] Jackson S.E., Maslach C. (1982). After-effects of job-related stress: Families as victims. J. Organ. Behav..

[17] Leiter M.P. (1988). Burnout as a Function of Communication Patterns. Group Organ. Stud..

[18] Maslach C., Jackson S.E. (1981). The measurement of experienced burnout. J. Organ. Behav..

[19] Rajesh J.I. (2016). The Level of Job Stress and Burnout Across Employees of Six Sectors in Indian Organizations. J. Organ. Hum. Behav..

[20] Liu Y., Lu L., Wang W.-X., Liu S., Chen H.-R., Gao X., Huang M.-Y., Liu Y.-N., Ren Y.-M., Wang C.-C. (2020). Job Burnout and Occupational Stressors among Chinese Healthcare Professionals at County-Level Health Alliances. Int. J. Environ. Res. Public Health.

[21] Panigrahi A. (2016). Managing stress at workplace. J. Manag. Res. Anal..

[22] Seňová A., Antošová M. (2014). Work Stress as a Worldwide Problem in Present Time. Procedia.-Soc. Behav. Sci..

[23] Halbesleben J., Buckley M.R. (2004). Burnout in Organizational Life. J. Manag..

[24] Omar M.K., Aluwi A.H., Fauzi M.W., Hairpuddin N.F. (2020). Work Stress, Workload, Work-Life Balance, And Intention To Leave Among Employees Of An Insurance Company In Malaysia. Int. J. Bus. Econ. Law.

[25] Rožman M., Treven S., Cingula M. (2018). The Impact of Behavioral Symptoms of Burnout on Work Engagement of Older Employees: The Case of Slovenian Companies. Naše Gospod./Our Econ..

[26] Anvari M.R.A., Kalali N.S., Gholipour A. (2011). Anvari_Kalali_Gholipour_2011_How does Personality effect on Job Burnout. Int. J. Trade Econ. Financ..

[27] Jiang H., Huang N., Jiang X., Yu J., Zhou Y., Pu H. (2021). Factors related to job burnout among older nurses in Guizhou province, China. PeerJ.

[28] Bianchi R., Truchot D., Laurent E., Brisson R., Schonfeld I.S. (2014). Is burnout solely job-related? A critical comment. Scand. J. Psychol..

[29] Jia L., Chudý S., Neumeister P., Bo Z. (2014). Study on the Job Burnout among University Teachers and Its Countermeasures. Scand. J. Psychol..

[30] Jiang X.-R., Du J.-J., Dong R.-Y. (2017). Coping Style, Job Burnout and Mental Health of University Teachers of the Millennial Generation. Eurasia J. Math. Sci. Technol. Educ..

[31] Maslach C., Jackson S.E., Leiter M.P. (1996). The Maslach Burnout Inventory Manual, The Maslach Burnout Inventory.

[32] Nabi Khan S. (2013). The Relationship between Job Burnout and Gender-Based Socio-Demographic Characteristics in Lahore. Lahore J. Bus..

[33] Wibowo A., Setiawan M., Yuniarinto A. (2021). The Effect Of Workloads On Turnover Intention With Work Stress As Mediation And Social Support As Moderated Variables. J. Apl. Manaj..

[34] Khafi A., Ghasemi M. (2014). The Relation between Job Burnout and Work and Family Conflict among Employees. J. Res. Dev..

[35] Makhdoom I.F., Atta M., Malik N.I. (2019). Counterproductive Work Behaviors as an Outcome of Job Burnout among High School Teachers. Bull. Educ. Res..

[36] Yousefy A., Ghassemi G.R. (2006). Job burnout in psychiatric and medical nurses in Isfahan, Islamic Republic of Iran. East. Mediterr. Health J..

[37] Inavalpotro J.A.S., Pérez M.S., Quiroga F.G. (2019). International Journal of Business and Social Science. Int. J. Bus. Soc. Sci..

[38] Ali N., Ali A. (2014). The Mediating Effect of Job Satisfaction between Psychological Capital and Job Burnout of Pakistani Nurses. J. Commer. Soc. Sci..

[39] Soelton M., Lestari P.A., Arief H., Putra R.L. (2019). The Effect of Role Conflict and Burnout Toward Turnover Intention at Software Industries, Work Stress as Moderating Variables. Adv. Econ. Bus. Manag. Res..

[40] Santhanam N., Srinivas S. (2019). Modeling the impact of employee engagement and happiness on burnout and turnover intention among blue-collar workers at a manufacturing company. Benchmarking Int. J..

[41] Roy S., Novak T., Miksaj-Todorovic L. (2010). Job Burnout among Prison Staff in the United States and Croatia: A Preliminary Comparative Study. Int. J. Crim. Justice Sci..

[42] Mojasa-Kajaob J., Golonka K., Marek T. (2015). Burnout And Engagement Among Teachers—Worklife Areas And Personality Traits As Predictors Of Relationships With Work. Int. J. Occup. Med. Environ. Health.

[43] Ali M., Bilal H., Raza B., Ghani M.U. (2019). Examining the Influence of Workplace Bullying on Job Burnout: Mediating Effect of Psychological Capital and Psychological Contract Violation. Int. J. Organ. Leadersh..

[44] Matin H.Z., Kalali N.S., Anvari M.R. (2012). Do Demographic Variables Moderate the Relationship Between Job Burnout and its Consequences?. Iran. J. Manag. Stud..

[45] Gil-Monte P.R., Olivares-Faúndez V.E., Jélvez-Wilke C., Mena L., Figueiredo-Ferraz H. (2014). Relationships between burnout and role ambiguity, role conflict and employee absenteeism among health workers. Ter. Psicol..

[46] Mardani S., Mardani N. (2014). The Impact of Psychological Empowerment on Job Burnout in Hospital Staff. Int. J. Hosp. Res..

[47] Beheshtifar M., Omidvar A.R. (2013). Causes to Create Job Burnout in Organizations’. Int. J. Acad. Res. Bus. Soc. Sci..

[48] Shah I.A., Fakhr Z., Ahmad M.S., Zaman K. (2010). Measuring Push, Pull and Personal Factors Affecting Turnover Intention: A Case Of University Teachers In Pakistan. Rev. Econ. Bus. Stud..

[49] Elçi1 M., Yildiz B., Karabay E. (2018). How Burnout Affects Turnover Intention? The Conditional Effects of Subjective Vitality and Supervisor Support. Int. J. Organ. Leadersh..

[50] Chen X., Ran L., Zhang Y., Yang J., Yao H., Zhu S., Tan X. (2019). Moderating role of job satisfaction on turnover intention and burnout among workers in primary care institutions: A cross-sectional study. BMC Public Health.

[51] Varghese S., Kumar J. (2019). Work Engagment and Burnout: A Study on Turnover Intention Among Educators. J. Xi’an Univ. Archit. Technol..

[52] Simon M., Müller B.H., Hasselhorn H.M. (2010). Leaving the organization or the profession—A multilevel analysis of nurses’ intentions. J. Adv. Nurs..

[53] Thomas J. (2013). Study on Causes and Effects of Employee Turnover in Construction Industry. Int. J. Sci. Res..

[54] Soelton M., Abadi Y., Gana N., Eko S., Putra T., Arief S., Haryanti D. (2020). Factors Affecting Turnover Intention Among Waiters In Franchise Restaurants’. South East Asia J. Contemp. Bus. Econ. Law.

[55] Abdou A.H., Khalil A.A.F., Mahmoud H.M.E., Elsaied M.A., Elsaed A.A. (2022). The Impact of Hospitality Work Environment on Employees’ Turnover Intentions During COVID-19 Pandemic: The Mediating Role of Work-Family Conflict. Front. Psychol..

[56] Xiaoming Y. (2014). Effects of Workload on Burnout and Turnover Intention of Medical Staff: A Study. Stud. Ethno-Med..

[57] Liu J., Zhu B., Wu J., Mao Y. (2019). Job satisfaction, work stress, and turnover intentions among rural health workers: A cross-sectional study in 11 western provinces of China. BMC Fam. Pr..

[58] Ahn J.-Y., Chaoyu W. (2019). Job stress and turnover intention revisited: Evidence from Korean firms. Probl. Perspect. Manag..

[59] Zahra S.S., Khan M.I., Imran M., Aman Q., Ali R. (2018). The relationship between job stress and turnover intentions in the pesticide sector of Pakistan: An employee behavior perspective. Manag. Issues Health Syst..

[60] Prasetio P., Partono A., Wulansari P., Putri S.T., Ramdhani R., Abdullah A. (2018). The Mediation of Job Satisfaction in the Relation of Work Stress and Turnover Intention in Hotel Industry. Adv. Econ. Bus. Manag. Res..

[61] Preacher K.J., Rucker D.D., Hayes A.F. (2007). Addressing Moderated Mediation Hypotheses: Theory, Methods, and Prescriptions. Multivar. Behav. Res..

[62] Huang S., van der Veen R., Song Z. (2018). The impact of coping strategies on occupational stress and turnover intentions among hotel employees. J. Hosp. Mark. Manag..

[63] Li X., Jiang T., Sun J., Shi L., Liu J. (2021). The relationship between occupational stress, job burnout and quality of life among surgical nurses in Xinjiang, China. BMC Nurs..

[64] Khalatbari J., Ghorbanshiroudi S., Firouzbakhsh M. (2013). Correlation of Job Stress, Job Satisfaction, Job Motivation and Burnout and Feeling Stress. Procedia.-Soc. Behav. Sci..

[65] Schaufeli W.B., Peeters M.C.W. (2000). Job Stress and Burnout Among Correctional Officers: A Literature Review. Int. J. Stress Manag..

[66] Henseler J., Ringle C.M., Sarstedt M. (2015). A new criterion for assessing discriminant validity in variance-based structural equation modeling. J. Acad. Mark. Sci..

[67] Maryati T., Kusumayuda A. (2020). Empirical Study of Job Stress, Turnover Intention, and Job Involvement -Study at PKU Muham7madiyah Hospital Yogyakarta. Adv. Econ. Bus. Manag. Res..

[68] Huynh Impacts of Job Stress and Dissatisfaction on Turnover Intention (2020). A Critical Analasys of Logistics Industry—Evidence from Vietnam. Int. J. Econ. Bus. Adm..

[69] Yukongdi V., Shrestha P. (2020). The influence of affective commitment, job satisfaction and job stress on turnover intention: A study of Nepalese bank employees. Rev. Integr. Bus. Econ. Res..

[70] Asheghi H., Asheghi M., Hesari M. (2020). Mediation Role of Psychological Capital Between Job Stress, Burnout, and Mental Health Among Nurses. Pr. Clin. Psychol..

[71] Sadeghipor N., Aghdam B.H., Kabiri S. (2021). Evaluation of Burnout and Job Stress in Care Worker and Comparison between Front-Line and Second-Line in Care Worker during Coronavirus Epidemic. Health Sci. J..

[72] Salem I.E.-B. (2015). Transformational leadership: Relationship to job stress and job burnout in five-star hotels. Tour. Hosp. Res..

[73] Hu H.-H., Cheng C.-W. (2010). Job stress, coping strategies, and burnout among hotel industry supervisors in Taiwan. Int. J. Hum. Resour. Manag..

[74] Wen B., Zhou X., Hu Y., Zhang X. (2020). Role Stress and Turnover Intention of Front-Line Hotel Employees: The Roles of Burnout and Service Climate. Front. Psychol..

[75] Meintjes A. (2019). Job stress and turnover intention of employees in the South African steel manufacturing industry—A management challenge. J. Contemp. Manag..

[76] Salvagioni D.A.J., Melanda F.N., Mesas A.E., GonzaÂlez A.D., Gabani F.L., Andrade S.M. (2017). Physical, psy-chological and occupational consequences of job burnout: A systematic review of prospective studies. PLoS ONE.

[77] Setyadi D., Paminto A., Defung F., Adhimursandi D. (2021). Workplace Incivility and Job Burnout and Work Engagement Effects on Turnover Intention of Coal Mining Company Employees in East Kalimantan. Int. J. Bus. Manag. Invent..

[78] Han S.J., Bonn M.A., Cho M. (2016). The relationship between customer incivility, restaurant frontline service employee burnout and turnover intention. Int. J. Hosp. Manag..

[79] Rahim A., Cosby D.M. (2016). A model of workplace incivility, job burnout, turnover intentions, and job performance. J. Manag. Dev..

[80] Kartono K., Hilmiana H. (2018). Job Burnout: A Mediation between Emotional Intelligence and Turnover Intention. J. Bisnis Dan Manaj..

[81] Uzuntarla Y., Bayer N., Golbasi Z., Akarsu K. (2021). Job satisfaction, burnout and turnover intention of nurses working in hospital during the pandemic COVID-19 in Turkey. J. Clin. Med. Kazakhstan.

[82] Chan C.M., Chang S.M., Chong Y.S., Tang C.U. (2015). The influence of job stress, burnout and job satisfaction among primary school teachers in Ipoh. E-Convers. Propos. A Clust. Excell..

[83] Kim H., Stoner M. (2008). Burnout and Turnover Intention Among Social Workers: Effects of Role Stress, Job Autonomy and Social Support. Adm. Soc. Work.

[84] Permarupan P.Y., Al Mamun A., Samy N.K., Saufi R.A., Hayat N. (2020). Predicting Nurses Burnout through Quality of Work Life and Psychological Empowerment: A Study Towards Sustainable Healthcare Services in Malaysia. Sustainability.

[85] Shanafelt T.D., Boone S., Tan L., Dyrbye L.N., Sotile W., Satele D., Oreskovich M.R. (2012). Burnout and satisfaction with work-life balance among US physicians relative to the general US population. Arch. Int. Med..

[86] Shreffler K.M., Meadows M.P., Davis K.D. (2011). Firefighting and Fathering: Work-Family Conflict, Parenting Stress, and Satisfaction with Parenting and Child Behavior. Father. A J. Theory Res. Pr. Men Father..

[87] Qattan A.M.N. (2017). The Effect of Work-Related Stress and Burnout on Nursing Performance and Job Satisfaction: A Study of Hospitals in Saudi Arabia. Ph.D. Thesis.

[88] Chiang C.-F., Liu B.-Z. (2017). Examining job stress and burnout of hotel room attendants: Internal marketing and organizational commitment as moderators. J. Hum. Resour. Hosp. Tour..

[89] Jung H.S., Yoon H.H., Kim Y.J. (2012). Effects of culinary employees’ role stress on burnout and turnover intention in hotel industry: Moderating effects on employees’ tenure. Serv. Ind. J..

[90] Rizzo J.R., House R.J., Lirtzman S.I. (1970). Role Conflict and Ambiguity in Complex Organizations. Adm. Sci. Q..

[91] Chromy J.R., Lavrakas P. (2008). Snowball Sampling. Encyclopedia of Survey Research Methods.

[92] Hair J.F., Black W.C., Babin B.J., Anderson R.E. (2010). Multivariate Data Analysis.

[93] Rodríguez-Ardura I., Meseguer-Artola A. (2020). Editorial: How to Prevent, Detect and Control Common Method Variance in Electronic Commerce Research. J. Theor. Appl. Electron. Commer. Res..

[94] Podsakoff P.M., MacKenzie S.B., Lee J.Y., Podsakoff N.P. (2003). Common method biases in behavioral research: A critical review of the literature and recommended remedies. J. Appl. Psychol..

[95] Chin W.W., Gopal A., Salisbury W.D. (1997). Advancing the Theory of Adaptive Structuration: The Development of a Scale to Measure Faithfulness of Appropriation. Inf. Syst. Res..

[96] Duckworth A.L., Kern M.L. (2011). A meta-analysis of the convergent validity of self-control measures. J. Res. Pers..

[97] Tziner A., Rabenu E., Radomski R., Belkin A. (2015). Work stress and turnover intentions among hospital physicians: The mediating role of burnout and work satisfaction. Rev. Psicol. Trab. Organ..

[98] Kelloway E.K. (1995). Structural equation modelling in perspective. J. Organ. Behav..

[99] Zhao X., Lynch J.G., Chen Q. (2010). Reconsidering Baron and Kenny: Myths and truths about mediation analysis. J. Consum. Res..

[100] Rehman N., Mubashar T. (2017). Job stress, psychological capital and turnover intentions in employees of hospitality industry. J. Behav. Sci..

[101] Babakus E., Yavas U., Karatepe O.M. (2008). The Effects of Job Demands, Job Resources and Intrinsic Motivation on Emotional Exhaustion and Turnover Intentions: A Study in the Turkish Hotel Industry. Int. J. Hosp. Tour. Adm..

[102] Cho J.-E., Choi H.C., Lee W.J. (2013). An Empirical Investigation of the Relationship Between Role Stressors, Emotional Exhaustion and Turnover Intention in the Airline Industry. Asia Pac. J. Tour. Res..

[103] Ahmad A., Afgan S. (2016). The Relationship of Job Stress and Turnover Intention in Commercial Banks of Pakistan by Assessing the Mediating Role of Burnout. J. Bus. Strateg..

